# Behavioral biases when viewing multiplexed scenes: scene structure and frames of reference for inspection

**DOI:** 10.3389/fpsyg.2013.00624

**Published:** 2013-09-24

**Authors:** Matthew J. Stainer, Kenneth C. Scott-Brown, Benjamin W. Tatler

**Affiliations:** ^1^Active Vision Lab, School of Psychology, University of DundeeDundee, UK; ^2^Department of Optometry and Vision Science, University of MelbourneMelbourne, VIC, Australia; ^3^Division of Psychology, University of Abertay DundeeDundee, UK

**Keywords:** scene viewing, scene structure, central bias, multiplex, frames of reference

## Abstract

Where people look when viewing a scene has been a much explored avenue of vision research (e.g., see Tatler, 2009). Current understanding of eye guidance suggests that a combination of high and low-level factors influence fixation selection (e.g., Torralba et al., 2006), but that there are also strong biases toward the center of an image (Tatler, 2007). However, situations where we view multiplexed scenes are becoming increasingly common, and it is unclear how visual inspection might be arranged when content lacks normal semantic or spatial structure. Here we use the central bias to examine how gaze behavior is organized in scenes that are presented in their normal format, or disrupted by scrambling the quadrants and separating them by space. In Experiment 1, scrambling scenes had the strongest influence on gaze allocation. Observers were highly biased by the quadrant center, although physical space did not enhance this bias. However, the center of the display still contributed to fixation selection above chance, and was most influential early in scene viewing. When the top left quadrant was held constant across all conditions in Experiment 2, fixation behavior was significantly influenced by the overall arrangement of the display, with fixations being biased toward the quadrant center when the other three quadrants were scrambled (despite the visual information in this quadrant being identical in all conditions). When scenes are scrambled into four quadrants and semantic contiguity is disrupted, observers no longer appear to view the content as a single scene (despite it consisting of the same visual information overall), but rather anchor visual inspection around the four separate “sub-scenes.” Moreover, the frame of reference that observers use when viewing the multiplex seems to change across viewing time: from an early bias toward the display center to a later bias toward quadrant centers.

## Introduction

The decisions about where to direct our small window of clear high-acuity vision in the world are tightly bound to both the task at hand (e.g., Buswell, 1935; Yarbus, 1967) and the contextual information provided by a scene (e.g., Torralba et al., 2006; Ehinger et al., 2009). Locations selected for gaze also tend to correlate with low-level features such as edges (Baddeley and Tatler, 2006). However, there are also prominent behavioral biases in the way that people move their eyes. For example, viewers show a strong tendency to bias distributions of fixations toward the center of the scene (Tatler et al., 2005; Tatler, 2007; Tseng et al., 2009) and toward the center of objects (Nuthmann and Henderson, 2010; Foulsham and Kingstone, 2013). These biases themselves provide insights into the manner in which scenes are viewed, suggesting that inspection behavior is organized around the conceptual units of objects and the scene.

The notion that inspection behavior is biased toward—and therefore organized around - the center of the scene is derived from experiments which use single static (Tatler, 2007) or dynamic (Tseng et al., 2009) scenes presented on computer monitors. However, even when engaged with computer displays, users rarely view single, full-screen images. Often, users are faced with multiple, windowed displays of visual information—and this multiplexed view of the world is increasingly common in news and entertainment media. Displaying visual information across multiple monitors or display windows introduces a level of organization that is not present in single-scene displays. Multiplex displays tend to include two types of disruption across the individual scenes. First, there is a discontinuity in the coherence of visual content across scenes and hence semantic continuity between scenes. Second, there is a physical discontinuity between scenes, often in the form of spatial separation between scenes in a multiplex or as abrupt line terminations between abutting scenes. It is not clear how these disruptions impact viewing behavior.

Specifically it is not clear whether scene-center biases found in single scene viewing will manifest as display-center biases, window-center biases or a mixture of these two when viewing multi-windowed displays. The relative importance of the display or window center when inspecting multiplexed displays can inform us about the extent to which multiplex scene viewing is organized around the entire multiplex display or the individual windows of the display (i.e., the “frame of reference” for inspection; Wade, 1992). Furthermore, we can consider whether manipulating the visual content of the display influences the relative importance of these two potential organizing centers for inspection.

### The central bias

The rules of photograph composition lead photographers to commonly bias their shots with interesting content in the center of the scene (Parkhurst and Niebur, 2003; Foulsham and Underwood, 2008). If scene viewing strategy is based solely around the interesting objects contained in a scene (e.g., Buswell, 1935; Yarbus, 1967; Nuthmann and Henderson, 2010; Foulsham and Kingstone, 2013), then the tendency for photographers to position objects of interest toward the center of images would consequently lead to a greater proportion of fixations in the central area. However, when Tatler (2007) examined images where the distribution of content was *not* biased toward the center (but rather the left side, for example), observers still showed a central bias in their fixation behavior. Of course, the mere existence of a photographer bias in itself might be the reason for the central bias, as observers might simply expect the most important content to be in the center of the scene. Thus, Parkhurst et al. (2002) suggested that with repeated exposure to images, this viewing strategy might have been developed as an efficient way of exploring and understanding images.

Vitu et al. (2004) found that by offsetting text from the screen center, an observer's first saccade would land near to the center of the word, but was also influenced by the location of the display center. It would therefore appear that both the viewed content, and the display center can contribute to fixation selection. However, when the screen was offset, fixation selection was influenced by both the content and the screen center, but there was no influence of the “straight-ahead” position. As there is an observed preference for making saccades to recenter the eye in its orbit (Fuller, 1996), this would suggest that the central bias appears to be responsive to the viewed content, rather than based wholly on an orbital reserve mechanism (as in Fuller, 1996; Carmi and Itti, 2006). This may, at least in part, be that in the absence of expectation about a scene's content, the center of a scene is the area of maximum information gain. During scene viewing, a preferential bias toward making fixations in the center of objects has been observed (Henderson, 1993; Nuthmann and Henderson, 2010; Foulsham and Kingstone, 2013; Pajak and Nuthmann, 2013). Consequently there has been shown to be a processing benefit to a more central fixation position (Foulsham and Kingstone, 2013), with objects being recognized faster when being presented with the center of the object being aligned to the observer's fixation, rather than shifted to the left or right of fixation. Thus, the center of a scene may be the area that will tell the viewer the most about the scene given that it is the location where most of the image will fall on the retina (albeit at decreasing acuity from the fovea).

In multiplex viewing, the center of the multiplex might offer an optimal viewing position for the entire display, but not for any individual scene within it: for these an optimal viewing position might be the center of each scene within the multiplex. As such, we can use the viewing biases of human observers to gain insights into how viewing behavior is organized when viewing a mutliplex. If the multiplex is viewed as a single, large scene then we might expect a tendency to fixate the center of the multiplex rather than the center of each display within the multiplex. However, if the multiplex is viewed as a collection of discrete scenes, we might expect fixations to cluster around the center of each scene within the multiplex. In this study we examine the influence of each of both semantic, and spatial disruption of content on how gaze is allocated across multiplex displays by scrambling scenes, and separating them by space.

### Semantic contiguity

Scenes in a multiplex often lack coherence and continuity of content between adjacent windows or panels, such as the multiple camera feeds displayed in a CCTV surveillance control room. Such discontinuity can also be introduced by taking a large single scene, dividing it into segments and randomly re-arranging them. This technique has been used previously on a number of occasions and is referred to as scrambling (Foulsham et al., 2011), jumbling (Varakin and Levin, 2008) and rearrangement (Sanocki et al., 2006). In this paper, we use the term “scrambling” to describe the method of shuffling sections of a scene. This manipulation to a single large scene essentially allows us to produce a multiplex that contains the same visual content as the original scene. We can therefore compare the original and shuffled versions of the scene in order to gain insights into the perceptual consequences of disrupting scene coherence in a multiplex.

Disrupting the structure of scenes by scrambling the contents has been previously shown to have a several consequences. First, search for objects and object recognition is poorer in scrambled scenes than in regular scenes (Biederman, 1972; Foulsham et al., 2011). Second, scrambling scenes leads to increased changes detection difficulty (Varakin and Levin, 2008). Third, perception of time is influenced by scrambling scenes, with participants subjectively rating scrambled scene presentation as significantly shorter than regular scene presentation (Varakin et al., 2013). Fourth, spatial representation is poorer in scrambled scenes (Sanocki et al., 2006) and fifth, scene structure influences eye movement behavior during scene viewing (Foulsham et al., 2011). An overriding message from these studies is that scene structure is used to guide the eyes, with targets being found faster, for example, when scene coherence is intact.

The structure of natural scenes tends to be governed by a set of underlying principles (such as that people tend to be located on the horizontal plane, and objects are acted on by natural forces such as gravity). Therefore we are able to use our prior knowledge about the world, and about the specific type of scene to determine that, for example, the best place to search for a clock is likely to be the walls (Henderson and Hollingworth, 1999). Torralba et al. (2006) demonstrated that such a contextual map of a scene can account for a great deal of where people allocate fixations (see also Ehinger et al., 2009), suggesting that scene structure plays a prominent role in scene inspection. In Torralba's model, low level features only moderately improve a model based on contextual cueing. However, when Foulsham et al. (2011) scrambled scenes, they found that low level features were much more prominent in fixation selection. This result implies that scrambling the scene, and thus disrupting the structural coherence between parts of the scene, removes the utility of knowing where objects are likely to be found, and changes the manner in which a scene is inspected. It further implies that the coherence of overall scene structure is central to using knowledge about expected spatial context rather than local spatial associations. In the present paper we consider similar issues by dividing a single large scene into quadrants and comparing viewing behavior for scrambled and original arrangements of the scene. However, as our index of viewing behavior we consider not the relative involvement of low level features in fixation selection, but rather the biases present in viewing behavior. This approach allows us to consider the relative biases toward the display and quadrant centers and thus the extent to which viewing behavior is organized around each of these two frames of reference in the multiplex.

### Spatial continuity

One of the consequences of scrambling scenes is the introduction of unnatural line terminators. For example, where the image of a car once continued across space, scrambling means that the front half of a car might now physically abut a wall. These physical discontinuities may in themselves result in changes to viewing behavior independently of or additionally to any effects introduced by disrupting the coherence of scene structure. In order to consider the relative impact of physical discontinuity and structural coherence in multiplexed scenes, we can introduce physical discontinuity but preserve coherent content between adjacent plexes. Introducing physical separation, or gridlines between segments of a scene can act to equalize the number of line terminators in original and scrambled versions of the scene. This physical separation has not been shown to influence scene viewing when semantic scene information is unimportant to the viewing task such as visual search (Tan and Czerwinski, 2003; Hutchings et al., 2004; Bi et al., 2010). However, Varakin et al. (2013) suggest that when scene context is important, that the physical separation of content may influence scene perception. An interesting question arises if we consider introducing space between quadrants of a scene but maintaining the normal arrangement of the scene. Scenes are frequently fragmented in this way in our everyday experience: as we look through the panes of a window or if we display images across multiple monitors. However, it is unclear how such forms of visual disruption to content might influence viewing behavior. One possibility is that the separation creates an additional frame of reference for each individual quadrant of the scene, thus the prediction would be that fixations are driven to these center points. Alternatively, they may be less intrusive to scene perception, since they might be parsed simply as the muntin grid of a window frame rather than a frame itself, and thus “seen through.” By using a non-occluded, separated view of the scene we create a stimulus similar to a bezeled array of monitors rather than a window, and so create the potential for additional frames of reference.

### The present study

A tendency to look in the center of pictures seems to be a characteristic trait of scene viewing (e.g., Tatler, 2007). Here, the central bias is used to examine what happens when scene structure is disrupted, as is often the case in multiplex displays. If viewing behavior is organized around the entire set of visual information presented on a monitor—effectively viewing the multiplex as a single large scene—then spatial allocation of gaze should not be influenced by whether this information contains coherent and continuous semantic and physical structure. We quantify the individual contributions of biases toward the center of the screen and of the center of each of the four quadrants on fixation selection. If semantic continuity is important for defining the boundaries of a scene for the purposes of organizing inspection behavior, then the prediction is that scrambling should lead to greater contribution of the quadrant center, and a decreased contribution of the screen center compared to the original version. Similarly, if spatial continuity is important then it follows that separating content by space should have the same effect on viewing behavior. Thus we not only consider the overall relative tendency to fixate near the center of the display or quadrant, but also whether this changes over the course of viewing the scenes for several seconds. Previous work has suggested that viewing behavior is different soon after onset than several seconds later (e.g., Buswell, 1935; Unema et al., 2005; Velichkovsky et al., 2005) and there is debate about whether this reflects differential reliance on low- and high-level factors (e.g., Parkhurst et al., 2002; Tatler et al., 2005, 2011). When viewing a scene the tendency to fixate the center of the scene diminishes over time (Tatler, 2007). These findings suggest that the factors that underlie fixation selection may vary over the course of viewing a scene and we therefore explore whether any tendency to fixate near the center of the scene or the center of a quadrant changes as viewing time progresses. In the present study we extend these ideas to consider whether biases toward the display or quadrant centers change over time and use this to infer whether the relative importance of these two frames of reference for organizing viewing behavior changes over time during multiplex viewing.

## Experiment 1

### Methods

#### Participants

The participants for this study were twenty-four undergraduate students (7 male, mean age 22.7 years). They took part in this experiment in exchange for course credit, and all reported normal vision.

#### Experimental apparatus

Images were prepared in Matlab (version 2007a), and presented on a CRT monitor with resolution of 1280 × 1024 and a refresh rate set at 85 Hz. Observers used a chin-rest to maintain head position at a viewing distance of approximately 60 cm. Eye position was recorded for the dominant eye using an Eyelink 1000 eye tracker, sampling pupil and corneal reflection at 1000 Hz. Saccades were detected using the SR Research algorithm with standard parameter settings.

#### Stimuli

**Figure 1:**
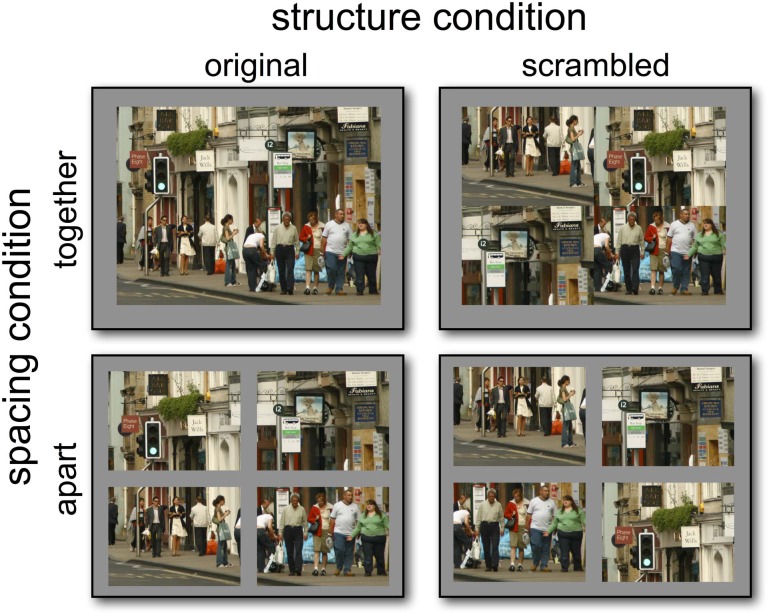
**Examples of stimuli from Experiment 1.** Stimuli were presented in one of two structure and spacing conditions: four quadrants abutting or spatially apart and four quadrants scrambled or normally configured.

Forty-eight target scenes were used in Experiment 1. These scenes were of four different types; city center, car park, airport terminal and traffic scenes. Each scene was presented either in one of two spacing conditions; touching or separated by space, and was presented in its original form, or with its quadrants in scrambled order (Figure 1). Scenes were presented at 800 × 600 pixels in size with each quadrant being 400 × 300 pixels. Stimuli that were separated had a 50 pixel bar between the quadrants. The scrambling of scenes was randomized in Matlab, although configurations were determined so that none of the originally continuous edges were touching. If the randomized configuration contained these consecutive edges, a new randomization would be carried out until a suitable configuration was produced. The arrangement of the quadrants in the scrambled conditions was randomized for each participant.

#### Procedure

Participants were asked to memories a series of 48 scenes for a memory task (which did not take place). Each scene was presented for 10 s, and participants were not told the organization or spacing information about each trial. Stimuli were counterbalanced so that the same image would occur in all four presentation styles an equal number of times across participants.

### Results

#### Analysis

Data were analyzed in a method based on Tseng et al. (2009) in *R* system for statistical computing (version 2.10.1; R Development Core Team, 2009). A random set of fixations was generated across the space which the scene subtended. The distance from the real and random fixations from the screen center, and the quadrant centers were calculated and compared. This allowed a calculation of the distance of each observed fixation from each potential center bias (the center of each quadrant and the center of each screen) *relative* to the mean random distance from each center, using the equation:

$$R_{dist}=100-(\frac{100}{MeanRand_{dist}})\times Obs_{dist}$$

wherein *R*_*dist*_ represents the difference in distance of observed fixations (*Obs*_*dist*_) from that expected for the random simulations, expressed as a percentage change in distance. Thus, fixations that are closer to the compared center than random will result in a positive *R_*dist*_* percentage, and fixations that are further will result in a negative *R*_*dist*_ percentage. This method allows us to examine the contribution of different biases (in this case the bias toward the center of each quadrant and the center of the screen) on fixation selection.

The relative contribution of the bias of the display center and quadrant center on each fixation was then compared using a linear mixed effect model. Linear mixed effect modeling has garnered increasing use in psychology as the method allows analysis of fixed and random factors (see Kliegl, 2007; Baayen et al., 2008). Whereby conventional statistical approaches such as Analysis of Variance condense each participant's data to a single cell mean, (meaning that individual variance contributes little to the overall pattern of data), linear mixed effect modeling allows all of the data to be used, controlling for random factors such as participant and stimuli (e.g., see Druker and Anderson, 2010). Here, we analyzed the data using the *lmer* function, in the *lme4* package (Bates, 2005) in *R* to calculate *t*-values (compared to a null model), with *p*-values calculated using the *pval.fnc* function in the *languageR* package (Baayen, 2008). Appropriate calculation of degrees of freedom for the *t*-value is still debated, and therefore not included (see in Druker and Anderson, 2010). *P*-values are estimated by generating model parameters using Markov Chain Monte Carlo (MCMC) sampling. We also present mean differences calculated with MCMC. Graphs were created using the *ggplot2* package in *R* (Wickham, 2009).

**Figure 2:**
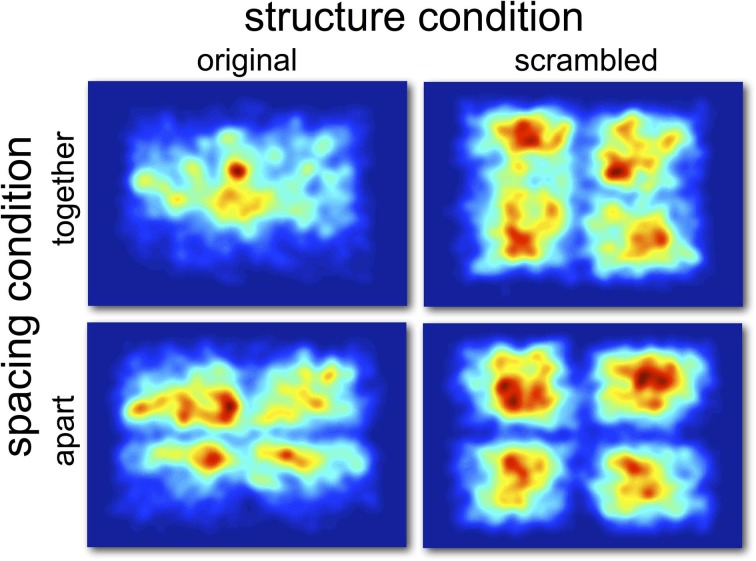
**Gaussian heat-maps of fixation distributions in Experiment 1.** A half degree kernel smoothing was applied to the gaussians.

**Figure 3:**
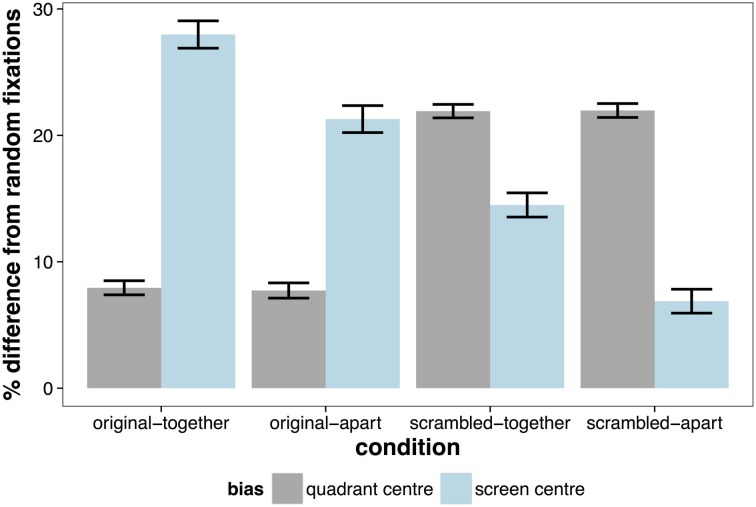
**Percentage improvement from random fixations for biases of the scene (quadrant) center and of the screen center.** Error bars indicate ± 1 SE.

***Contribution of quadrant center and screen center biases***. Figure 2 shows the pattern of fixations in Experiment 1. A Linear Mixed Effect Model (with participant, scene and ordinal trial number entered as random effects) revealed that there was a highly significant effect of scene organization on the distance from the center of each quadrant (*t* = 35.94, *p* < 0.001), with scenes that were scrambled having fixations that were 14.67% closer to the quadrant center (Figure 3). There was no significant effect of spacing (*t* = 1.80, *p* = 0.68).

**Figure 4:**
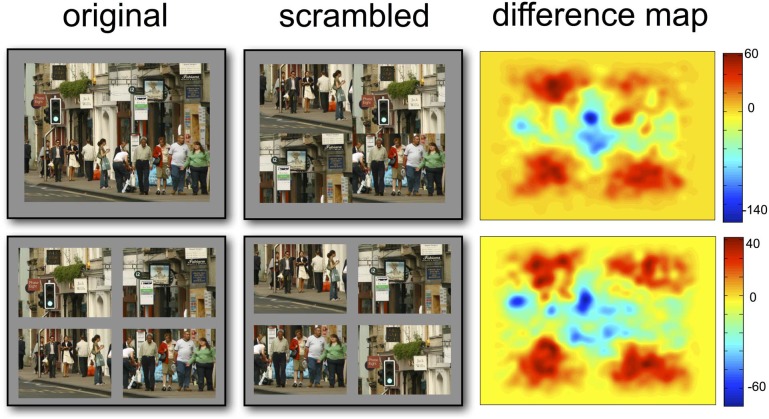
**Visualization of differences in fixation distributions between original and scrambled scenes.** Red areas indicate areas of higher fixation numbers in the scrambled versions of the scenes.

The opposite effect was observed when considering the distance of fixations from the screen center, with fixations being significantly more central in the original presented format (MCMC mean = 14.5%, *t* = 35.80, *p* < 0.001). There was also a significant effect of whether the scene was separated by space, which was on average 8.29% closer to the screen center in together condition (*t* = −20.27, *p* < 0.001)^1^. The pattern of results is summarized in Figure 3, which shows the percentage difference in distance from the screen or quadrant center from the distances of a random set of fixations. Values greater than zero suggest a greater-than-random tendency to fixate close to the screen/quadrant center, with higher values indicating stronger central tendencies. The screen center appears to have an influence in all conditions, but is much more important when the scene is in its original format (see Table 1). However, when scenes are scrambled, the bias toward the center of the quadrant is more influential than the screen center. Figure 4 shows differences in the spatial distribution between scrambled and original versions of the scenes for both spatial organization conditions.

**Table 1:** **Summary of mean improvement from random of quadrant and screen biases in each condition with linear mixed effect *t*-values**.

**Condition**	**Quadrant mean**	**Screen mean**	***t*-value**
(1) Original—together	8.88	28.54	36.95***
(2) Original—apart	8.40	20.99	21.62***
(3) Scrambled—together	23.05	15.13	−16.32***
(4) Scrambled—apart	21.93	7.71	−27.09***

*All tests were significant at the p < 0.001 level*.

**Figure 5:**
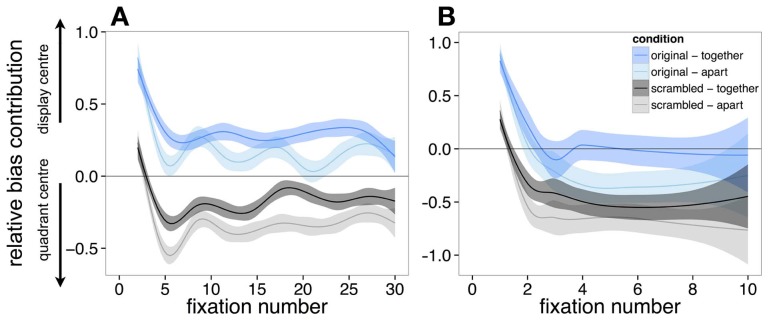
**The relative contribution of the quadrant center, and display center on fixation selection when considering (A) all fixations, and (B) only the fixations that landed on the first quadrant that was inspected.** Bias toward the display center is at 1, with bias toward the quadrant center bias at −1. Standard Errors (±1) are shaded. As the lines move toward the ±1, this represents a stronger contribution of the bias to fixation selection.

***Time-course of central biases***. The contribution of both the display center and quadrant center was examined across each fixation in each trial. However, one consideration was in the manner in which best to index fixation. To demonstrate this problem, Figure 5A reveals that when we consider all fixations on every trial. While all conditions initially are biased by the display center (on the first fixation after onset), this quickly changes in scrambled scenes to be more biased toward the quadrant center. However, the lines are relatively wavy. One possible cause of this apparent waviness is that it represents relocation to different quadrants at different times during viewing. Accordingly, Figure 5B presents only the fixations from the first inspected quadrant. In presenting this data, there are fewer data points for each ordinal fixation (as evidenced by larger error shading compared to Figure 5B), but smoother curves. While considering fixations only in the first quadrant may not be as relevant for the scenes that are presented in their original format (based on the null bias toward the quadrant centers), we present the data in this way for completeness.

**Figure 6:**
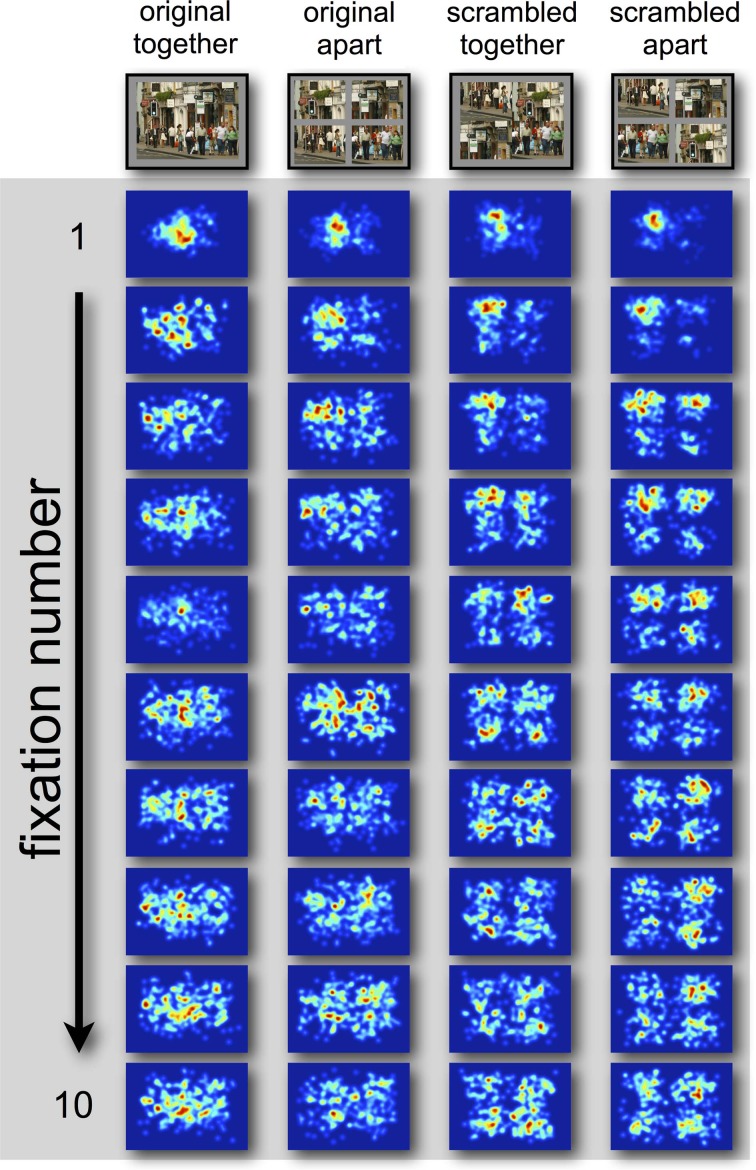
**Gaussian heat-map of the time-course fixation distribution by ordinal fixation number**.

Regardless of the visualization style of the data, the pattern is relatively consistent. The landing position of the first saccade in all display types is consistently biased toward the display center (Figures 5A,B). After this fixation, viewing strategy changes in scrambled scenes, with observers' fixations being biased by the quadrant center significantly more than the display center, which drops to around chance performance (0% different from random) in the condition where the scene is scrambled and separated by space. This likely represents more peripheral exploration from the coherent scene center, as is common in scene viewing (Tatler, 2007). In support of this, Figure 6 shows a heat-map visualization of fixation distribution by ordinal fixation number.

### Discussion

In Experiment 1 we recorded observers' eye movements during a picture memorizing task where we systematically manipulated the arrangement and separation of the four quadrants of the source images. We found that when scene structure was disrupted by scrambling, fixations tended to be more biased toward the center of each scene quadrant than when the scenes were not scrambled. This was found irrespective of whether the quadrants were touching or separated by a gap between them. One interpretation of these data is that scrambling the scenes results in a viewing strategy that is based around scene quadrants rather than viewing the display as a whole. If the central bias is an indicative feature of how we view scenes, this would correspondingly imply that the viewer essentially behaves more like they are viewing four separate scenes when faced with a scene that has been scrambled into quadrants. This suggests that continuity of content between parts of a scene, rather than the semantic content within each part, is essential for how we understand scenes and their boundaries (also Foulsham et al., 2011; Varakin et al., 2013).

It also appears from these findings that scrambling to disrupt the continuity of information between quadrants has a greater effect on viewing behavior than physically separating the quadrants of the scene. The lack of difference in quadrant center bias between the touching and separated physical layouts of the scenes suggests that separation alone does not result in a greater tendency to base viewing behavior around the centers of the individual scene quadrants. As such, there is no evidence that physical separation changes our understanding of the scene in this way. It would appear from these findings therefore that disrupting the semantic continuity across scene quadrants results in viewing behavior that is more based around the quadrant, whereas physical separation does not. Thus perhaps a lack of continuity of content between neighboring parts of a display is a cue for identifying the boundary of a scene whereas we can tolerate physical interruption to a scene as long as the continuity between neighboring (but separated) parts of a scene is maintained. These findings suggest that the frame of reference around which viewing is organized is defined not solely by physical separation between areas of visual content, but by whether neighboring quadrants have continuity of content. When there is continuity of content, the frame of reference for viewing appears to be the entire display; when continuity of content is disrupted by scrambling, the individual quadrants provide the key frames of reference for inspection (Wade, 1992).

The time-course of scene and quadrant center biases suggests that the tendency to look around the center of the display is most prominent at the start of viewing and diminishes over the first few fixations on the display. This findings is consistent with previous reports that viewing behavior changes over time when viewing single scenes (Buswell, 1935; Parkhurst et al., 2002; Tatler et al., 2005) and that the tendency to fixate near the center of the scene also reduces over time when engaged in a search task (Tatler, 2007). Furthermore this result implies that there may be a change in how the display is understood by the observer over time. Soon after onset, viewing is based around the center of the display irrespective of the arrangement in our Experiment. This is consistent with the observer viewing the entire display as a single scene. However, after several fixations, there is no longer a tendency to fixate the screen center more that the quadrant center, and if the quadrants are scrambled a the tendency to fixate near the quadrant center dominates. This is consistent with participants no longer viewing the entire display as a single scene, but viewing the display as a set of scenes.

One possible explanation for the pattern of results in Experiment 1 might be related to the fact that the images used are likely to contain photographer bias, with “interesting” scene elements in the center of the image. When viewing scenes, the visual system appears to select objects for inspection (preferentially landing near the center of the object; Nuthmann and Henderson, 2010; Foulsham and Kingstone, 2013). If the average distribution of objects in the scenes in Experiment 1 was indeed biased to the scene center, this would mean that the distribution of objects and features in a particular quadrant of the display would not be equivalent across our two structural arrangement conditions. For example, for scenes that are not scrambled, the top left quadrant of the display would on average contain a bias of content toward the lower right corner of that quadrant. However, in the scrambled condition, across all scenes content from all four possible quadrants of the source images are equally likely to be presented in the upper left of the multiple display. For these scenes the average distribution of content is unlikely to be biased toward the lower right of the quadrant and may be more uniformly or even centrally biased. It is therefore possible that the differences we found between original and scrambled arrangements of the scenes are confounded by these differences rather than reflections of different organization of inspection behavior.

Experiment 2 addresses this potential confound by keeping the information in the upper left quadrant the same for all conditions, only scrambling the other three scene quadrants. Therefore, the upper left quadrant is the same in all conditions, and any differences in viewing biases observed would be due to the arrangement of the other three quadrants. If the biases in viewing behavior for a particular quadrant are driven by information distribution within the viewed quadrant, we should expect to see the same distributions of fixations in the upper left quadrant when the entire scene is presented unmodified and scrambled. However, if there is a greater tendency to look toward the center of the quadrant when the scene is scrambled, as was found in Experiment 1, then this will suggest that scrambling the scenes promotes a quadrant-centred inspection behavior irrespective of information distribution within the quadrant (as in Tatler, 2007).

## Experiment 2

The aim of Experiment 2 was to ensure that the findings of Experiment 1 were not the consequence of the averaging of positions of objects and features across images. As objects appear to be a unit around which visual inspection is anchored (Nuthmann and Henderson, 2010; Foulsham and Kingstone, 2013), it is possible that the results from Experiment 1 were due to the differences in the distribution of objects in each quadrant that was created when the scene was scrambled. In Experiment 2, we used an identical procedure to Experiment 1 except that the top left quadrant of the image was always presented in its correct location. Thus, when we examine the distribution of fixations in the top left quadrant, any differences must necessarily arise from the arrangement of the other quadrants.

If the bias toward the center of a quadrant is simply a consequence of the scrambling of distributions of objects, and does not represent a true bias present in multiplex viewing then we would predict that when the left quadrant is held constant, fixations in this quadrant would not be influenced by the structure of the display. However, if the results of Experiment 1 represent a quadrant-based inspection bias in multiplex viewing, then we would predict that the distribution of fixations in the top left quadrant would be more biased toward the quadrant center when the other quadrants are scrambled. This can tell us about whether the structure of the quadrant, or the structure of the display is most influential in fixation selection when viewing multiplexed scenes.

### Methods

#### Participants

Twenty four students (9 males, average age 23.8 years) took part in Experiment 2 in exchange for course credit. They had not participated in the previous experiment and reported normal or corrected-to-normal vision.

#### Stimuli

Stimuli were the same as used in Experiment 1. The difference was that the random organization of the scrambled versions of the images always had the top left corner presented in its correct position. The arrangement of the non-constant quadrants in the scrambled conditions was randomized for each participant.

#### Procedure

The procedure was identical to the Experiment 1, except for the stimuli change described above.

### Results

**Figure 7:**
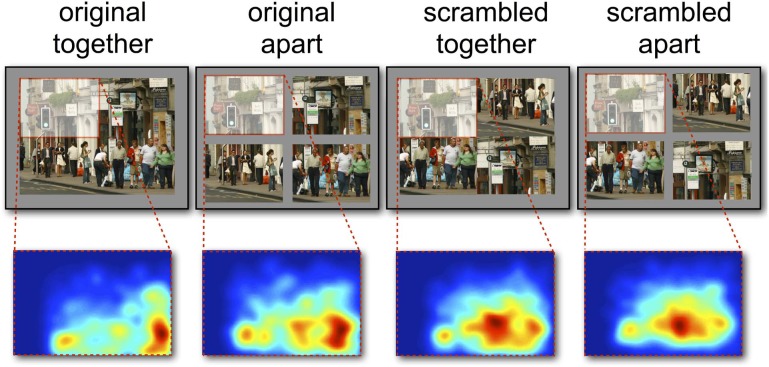
**Fixation distributions in the top left quadrant of scenes, with the area examined show in the top panel**.

**Figure 8:**
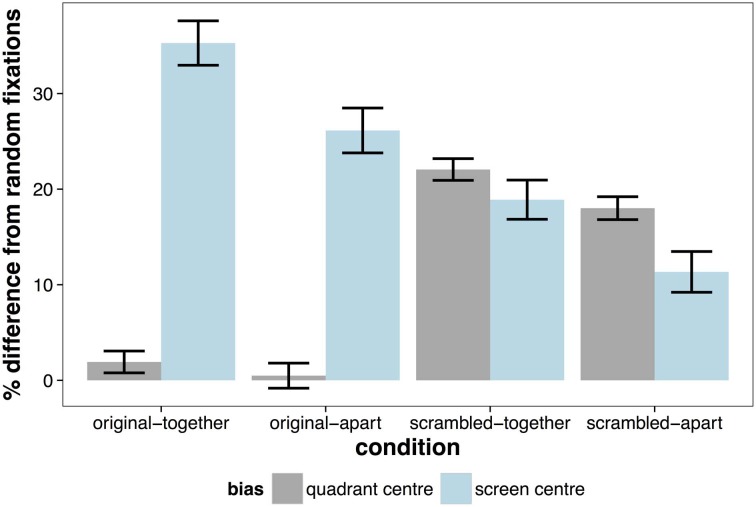
**Percentage improvement from random fixations for biases of the scene (quadrant) center and of the screen center in the top left quadrant.** Error bars indicate ±1SE.

The distribution of fixations in the top left quadrant of all trials is shown in Figure 7. There was a highly significant effect of scene organization, with fixation being 18.74% closer to the quadrant center in the scrambled scenes compared to the original scenes (*t* = 23.53, *p* < 0.001). There was also a significant effect of physical separation (*t* = 2.16, *p* < 0.001). Fixations were more biased toward the quadrant center in scenes that were separated by space, but this effect was modest (MCMC mean = 2.99%). Figure 8 reveals that there was no difference in the bias toward the quadrant center between a random set of fixations and fixations to scenes presented in their original format. When the quadrants of scenes are scrambled, there is a strong bias toward fixating the center of the quadrant.

The effect of organization and space was also highly significant in accounting for the distance of fixations from the screen center, but as in Experiment 1, this was in the opposite direction of the quadrant bias. Fixations were much closer to the screen center in scenes presented in original format than scrambled (MCMC mean = 15.4%, *t* = −19.72, *p* < 0.001). Correspondingly, fixations tended to be closer to the screen center when scenes were not separated with space (MCMC mean = 8.5%, *t* = 10.13, *p* < 0.001). The differences between the contribution of each bias in each condition was the same as Experiment 1, except that there was no significant difference between the contribution of the screen center and the quadrant center in the scrambled-together condition (*t* = −0.73, *p* = 0.47). The time-course of the influence of these biases in Experiment 2 replicated the findings of Experiment 1.

## General discussion

We conducted two experiments examining the contribution of biases toward the display center, and quadrant center on fixation locations during scene memorization when scenes were scrambled, and separated by space. When the scene was presented unmodified, fixation allocation was biased toward the center of the display and only very slightly biased toward the centers of each quadrant. This is as would be expected from previous reports of screen center biases in fixation allocation (Tatler, 2007; Tseng et al., 2009). However, when scene structure was disrupted by scrambling the quadrants of the scene, the tendency to fixate in the center of the screen was reduced (although not completely removed), and there was a tendency to fixate closer to the center of each individual scene quadrant than would be expected by chance.

Experiment 2 revealed that when the distribution of visual content in one of the quadrants was held constant, the arrangement of the other quadrants in the display influenced fixation selection. In displays where the non-constant quadrants were shuffled, fixations were distributed near to the center of the constant quadrant. The results of Experiment 1 do not therefore appear to arise from averaging the visual information across the quadrant when scenes were scrambled. In Experiment 2 there was also an effect of physical separation on fixation distributions, with scenes that were separated by space having fixations significantly further from the screen center (however, this effect was modest, and was not significant in Experiment 1).

### Frames for visual inspection

In the developed world, people are increasingly presented with visual information that is segmented across windows or displays. The results from the two experiments reported here suggest that under conditions where information in a visual display lacks semantic contiguity or spatial continuity between quadrants, the frames within which people organize how they allocate gaze are unlike those for undisrupted scenes. This is evidenced by a significant change in the relative contribution of screen- and quadrant-center biases when viewing scenes that are scrambled, and separated by space compared to undisrupted scenes. In particular, we found evidence that when scenes are scrambled, fixation allocation is more biased toward the quadrant center than toward the screen center. This finding is consistent with a change in the manner in which scene inspection is organized. When viewing single scenes, there is a prominent bias toward the center of the display, suggesting that viewing is in some way organized around this reference point, and it has been suggested that organizing inspection around this location might provide advantages for information-gathering or efficient exploration (Tatler, 2007; Tseng et al., 2009). However, here we show that when the content of the scene quadrants are scrambled, there is a clear bias toward the center of each of the four quadrants of the display. This suggests that in multiplex viewing, inspection might be organized around the centers of each window or panel in the multiplex. However, it should be noted that although scrambled the content of the display quadrants seems to be associated with the introduction of a bias of fixations toward quadrant centers, there is still evidence of a continued, but less prominent, bias toward the display center (the center of the entire quadraplex of quadrants; see for example Figure 3). As such, it would appear that the center of each window or panel of a multiplex and the center of the entire multiplex may both provide points of reference around which inspection behavior is organized. A similar joint influence of multiple frames of reference was found by Vitu et al. (2004) for the landing position of fixations within words. When isolated words were presented off-center on a display, observers landed near to the center of the word, but remained somewhat biased toward the display center.

We are not the first to have considered how dividing a scene into component parts and shuffling their organization influences viewing behavior. Foulsham et al. (2011) showed that when this manipulation is made (although they divided their scenes into more parts than in the present experiments) inspection behavior changed in several ways, including initial saccade latency, the number of fixation required to find a search target, and the average fixation duration. Moreover, the association between fixation selection and low-level visual features (visual salience) in the scene changed. For scrambled scenes, there was a stronger correlation between low-level visual features and fixation selection. This result suggests that when viewing a display containing windowed information that lacks coherent structure between windows, low-level image features may have a greater influence on inspection behavior than when viewing structurally coherent displays. If we combine the implications of Foulsham et al.'s (2011) study and those of the present study, we can suggest that when an observer is faced with a multiplexed display, the principles underlying fixation selection are considerably different from those underlying single scene viewing, with a greater involvement of low-level image features and a shift toward organization of inspection behavior around the centers of each window or panel of the display rather than the center of the entire multiplex display. However, in the present study we also considered whether and how the biases toward screen- and quadrant-centers changed over the time-course of viewing a multiplexed scene and found that the relative importance of these two biases changed considerably over time. These changes provide further insights into the strategy that observers might be using when they look at multiplex-like displays.

### Early influence of screen centre on gaze

The early stage of scene viewing appears to serve to parse an image into relative constituent parts (such as objects; Foulsham and Kingstone, 2013). However, the multiplex introduces a further layer of complexity by nesting many scenes within a display. Both the quadrant centers and the display center seemed to play a role in inspection behavior in our two experiments. However, examining the time-course of inspection behavior revealed that the relative prominence of screen- and quadrant-center biases changed as viewing progressed. Immediately after scene onset, the screen center appeared to be the main contributor to fixation selection, with fixations being close to the screen center irrespective of whether the quadrants were physically separated or not and whether the visual content was shuffled or not. Tseng et al. (2009) similarly found that influence of the screen center on fixation selection lasts for the first two saccades after a scene change. It is possible that our findings indicate an initial global parsing stage of inspecting a display during which inspection behavior is organized around the entire display irrespective of whether the viewed information is a single large scene or a set of physically separated windows with disrupted continuity of content between windows. This period soon after display onset may serve to determine the most appropriate manner in which to organize inspection behavior thereafter: either around the entire display if the scene is coherently structured, or around the constituent parts of a multiplexed display. The results of the present experiments suggest a change in the strategies underlying inspection behavior as viewing time progresses when viewing a multiplexed display, and the frames of reference around which viewing is organized, from a globally-organized inspection strategy soon after onset to a locally-organized strategy as viewing progresses. Similar suggestions of global followed by local processing have been suggested for scene and pattern perception in previous studies.

Navon (1977, 1981) demonstrated that when complex stimuli consist of global and local components (for example, a large latter H made of small Ss), that response times were faster for when people were asked to attend to the global structure (the large H) compared to when they were asked to attend to the local structure (the small Ss). As such, this “global precedence” would support the view that there might be two modes of viewing. Early in viewing, we would therefore expect that the global structure of the multiplex would have the strongest influence on gaze allocation.

Much of the meaning of a display (at least in single scene form) can be extracted extremely rapidly (~100 ms; e.g., Potter, 1975). As the first saccade landing position in our experiments was most influenced by the display center in all conditions, this initial fixation might reasonably be considered to be the result of an attempt to understand whether the scene does, or does not contain coherent natural structure (and therefore identify the type of display that is being viewed). When viewing scenes, there is growing evidence that inspection behavior is different soon after scene onset compared to inspection behavior after prolonged viewing. Observers tend to select similar locations to fixate soon after the appearance of a scene, but there is much less consistency in spatial selection after prolonged viewing (Buswell, 1935). It has been suggested that these differences reflect differential reliance on low-level and high-level factors in driving fixation selection, with a greater influence of low-level properties on fixation selection soon after scene onset than later on (e.g., Parkhurst et al., 2002). However, others have argued against this interpretation, suggesting that the contribution of low-level factors to fixation selection is unlikely to change over time, but rather the observed divergence in viewing behavior between individuals reflects a divergence in higher-level strategic factors (Tatler et al., 2005, 2011). Such change in strategy would of course be beneficial when interpreting whether the information in a display contains the aforementioned structure of a natural scene, and in instances where it does not; thus adopting a more segment-based viewing strategy.

Not only are there overall differences in where fixations are allocated in scenes as viewing time progresses, but there is also evidence that there may be systematic differences in processing styles within fixations soon after onset from that within later fixations (e.g., Velichkovsky et al., 2005). The suggestion is that soon after scene onset processing within fixations is primarily “ambient,” gathering global scene properties, whereas later in scene viewing processing within fixations become predominantly “focal,” gathering local information about objects. Velichkovsky and colleagues suggest that these two modes of viewing can be differentiated based on eye movement metrics: ambient processing is associated with large amplitude saccades in combination with short duration fixations; focal processing is associated with short amplitude saccades in combination with long duration fixations (Unema et al., 2005). Over et al. (2007) discuss viewing strategy as being “coarse-to-fine” in a visual search task, with saccade amplitude decreasing and fixation duration increasing across search time. This is thought to represent the early and late stage strategy of visual information processing (Pannasch et al., 2008).

### The central bias

The pattern of results found in Experiment 1 could reflect strategic differences associated with whether the display is understood as a single scene or set of smaller scenes by the observer. Alternatively the results might arise from the distribution of visual information within and between quadrants in the images, that reflects the typical “photographer bias” to place information at the center of the image. However, when the distribution of visual content within a quadrant is controlled in Experiment 2, photographer bias cannot account for the tendency to fixate closer to the center of the quadrant when the scene is scrambled or separated physically (as would be in line with Tatler, 2007). The bias toward the quadrant center was influenced not by the content in that quadrant, but by the overall arrangement of information in the display. Thus, the results presented in this paper suggest that the central bias is responsive to the viewed content (also Vitu et al., 2004). If the central bias was independent of scene structure, we would have expected that the center of the display would have the strongest influence on fixation selection in all conditions. However, there was a clear shift to a bias toward the center of the quadrant when content is scrambled. This would suggest that central fixation might reflect a learned expectation of “interesting” content being presented in the center of scene quadrants combined with a viewing strategy in which each quadrant is treated as a separate scene (Parkhurst et al., 2002). A second possibility is that the quadrant center is a convenient location to explore the content, as it minimizes the maximum distance the eye would have to travel within that scene to land on any possible location.

### Separation and occlusion

In the experiments presented here, we physically separated the quadrants by moving them apart. This reproduces the format of most multiple-scene displays, which terminate content at the monitor edge, and continue that content at the edge of the bordering display with the bezel separation in between. Under these viewing conditions, we find only modest influences of spatial separation on fixation selection. This finding is consistent with a similar lack of disruptive effect had previously demonstrated in different tasks (e.g., in visual search; Tan and Czerwinski, 2003). Of course, separation of our natural visual world into segments is not solely limited to the visual displays that we look at. Perhaps more common to our experience of the world are the physical occlusions provided by windows, whereby content continues to exist behind the occluding window pane.

In our experiments, we did not use occlusions, since to do so would mean occluding different visual information in the normal and scrambled scenes and that visual content that might itself be important for fixation allocation would not be seen by participants in the occlusion conditions. However, Varakin and Levin (2008) found that introducing occluding boundaries in a scene did not seem to influence viewing. They found that scrambling scenes reduced mean change detection performance, but introducing occlusions over the content (to equate the number of line terminations caused by scrambling) had no additional disruptive effect. In our experiments, when scenes were presented in their original format (the quadrant contents were not scrambled) but separated by space, people did appear to look to the spaces between scenes (e.g., see Figure 3). This might indicate some degree of attempting to reconcile the physical interruptions to the scene content. When scenes were scrambled and separated by space, the tendency to look at the spaces between scenes was not as pronounced. Thus, the overall structure of a scene (or the corresponding line endings between quadrants) seems to provide the observer with information that allows them to view a separated (but structurally normal) scene in a more holistic manner. Perhaps then, spending our lives looking through windows affords some ability to resolve information across space when a scene contains coherent structure^2^. Of course, it may be that the reason that we do not find a large effect of space is that the physical separation in this instance was too small. It may be that at larger sizes of spatial separations, people find it harder to consider a separated scene as a single percept (at least based on simple gestalt proximity grouping principles).

## Conclusions

Gaze allocation in single scenes is relatively well studied, with the locations that people look being influenced by a combination of high-level factors (e.g., Buswell, 1935; Yarbus, 1967), low-level factors (e.g., Torralba et al., 2006) and viewing biases. In particular, when people look at single scenes on a computer screen, there is a prominent central bias (Tatler, 2007) and this can explain much of where people look (Vincent et al., 2009; Tseng et al., 2009). However, users are increasingly presented with more complex displays, where visual content lacks the physical and semantic coherence of single scenes. Here, we examined what these biases can tell us about the way in which people arrange their viewing strategy when looking at such displays. When scenes are scrambled into four quadrants and semantic contiguity is disrupted, observers no longer appear to view the content as a single scene (despite it consisting the same visual information overall), but rather anchor visual inspection around the four separate “sub-scenes.” Disrupting a scene therefore appears to change the frames of reference that the observers use to inspect the display. Moreover, the frame of reference that observers use when viewing the multiplex seems to change across viewing time. Early in viewing, this frame appears to be the entire display (with fixations heavily influenced by the screen center). However, across time scrambling scenes leads to a change in fixation distribution toward the four quadrant centers. This paints a remarkably flexible picture of the frame of reference around which viewing is organized, suggesting that it may dynamically change to suit the viewing requirements of the displayed content. Foulsham et al. (2011) demonstrated that the correlation of low-level features at fixation is higher when scene structure is disrupted. Taken together, these results suggest that the underlying principles that govern eye guidance in scene perception are sensitive to scene structure. The relative combination of factors that influence where people look in scrambled scenes seems to be different to the factors that influence gaze selection in a single scene. Scene context plays an important role in where we look. We have demonstrated that when looking at a quadrant of an image, the context in which that quadrant is presented in (a normal, or scrambled scene) changes the way people allocate their gaze, despite the quadrant itself containing exactly the same information in all conditions. For any theoretical or empirical consideration of the role of context in scene viewing, it is important to take into account not only the contextual relationships within a scene, but also the relationships between windows in a multi-windowed display. Thus, the way that the observer segments visual information presented on a display into a scene is of crucial importance for our understanding of scene inspection and perception, particularly in light of the increasing prevalence of displays that contain more than one windowed scene.

## Ethics statement

This research was carried out in accordance with, and approval of the University of Dundee Ethics Committee.

### Conflict of interest statement

The authors declare that the research was conducted in the absence of any commercial or financial relationships that could be construed as a potential conflict of interest.

## Footnotes

^1^One potential problem with the comparison of space on the screen centre is whether we consider the centre of the image being the screen centre, or the central-most corner of the quadrants. However, when these two different reference points for calculating screen-centre biases were compared, there was no significant difference (*p* > 0.05).

^2^Looking through window frames is ecologically very similar to the occlusions provided by branches of trees. In both these conditions, the experimental re-creation of these images potentially can create an additional cue conflict with binocular viewing where minute differences in image content would be present in the natural condition but not in the screen based condition. Head movement produced parallax cues are an additional depth and interpretative cue available in natural viewing but not available in laboratory screen viewing.
